# Iodine-related attenuation in contrast-enhanced dual-energy computed tomography in small-sized solid-type lung cancers is associated with the postoperative prognosis

**DOI:** 10.1186/s40644-020-00368-1

**Published:** 2021-01-07

**Authors:** Shingo Iwano, Shinichiro Kamiya, Rintaro Ito, Shota Nakamura, Shinji Naganawa

**Affiliations:** 1 Department of Radiology, Nagoya University Graduate School of Medicine, 65 Tsurumai-cho, Showa-ku, Nagoya, 466-8550 Japan; 2 Department of Thoracic Surgery, Nagoya University Graduate School of Medicine, 65 Tsurumai-cho, Showa-ku, Nagoya, 466-8550 Japan

**Keywords:** Contrast enhancement, Dual-energy CT, Functional imaging, Iodine-related attenuation, Lung cancer

## Abstract

**Background:**

To investigate the correlation between iodine-related attenuation in contrast-enhanced dual-energy computed tomography (DE-CT) and the postoperative prognosis of surgically resected solid-type small-sized lung cancers.

**Methods:**

We retrospectively reviewed the DE-CT findings and postoperative course of solid-type lung cancers ≤3 cm in diameter. After injection of iodinated contrast media, arterial phases were scanned using 140-kVp and 80-kVp tube voltages. Three-dimensional iodine-related attenuation (3D-IRA) of primary tumors at the arterial phase was computed using the “lung nodule” application software. The corrected 3D-IRA normalized to the patient’s body weight and contrast medium concentration was then calculated.

**Results:**

A total of 120 resected solid-type lung cancers ≤3 cm in diameter were selected for analysis (82 males and 38 females; mean age, 67 years). During the observation period (median, 47 months), 32 patients showed postoperative recurrence. Recurrent tumors had significantly lower 3D-IRA and corrected 3D-IRA at early phase compared to non-recurrent tumors (*p* = 0.046 and *p* = 0.027, respectively). The area under the receiver operating characteristic curve for postoperative recurrence was 0.624 for the corrected 3D-IRA at early phase (*p* = 0.025), and the cutoff value was 5.88. Kaplan–Meier curves for disease-free survival indicated that patients showing tumors with 3D-IRA > 5.88 had a significantly better prognosis than those with tumors showing 3D-IRA < 5.88 (*p* = 0.017).

**Conclusions:**

The 3D-IRA of small-sized solid-type lung cancers on contrast-enhanced DE-CT was significantly associated with postoperative prognosis, and low 3D-IRA tumors showed a higher TNM stage and a significantly poorer prognosis.

## Introduction

The prognosis of primary lung cancer is affected by many clinical, imaging, and histopathological findings. In general, small-sized lung cancers with a diameter equal to or less than 3 cm have a good postoperative prognosis [1, 2]. In addition, the ratio of solid component and ground-glass opacity within a nodule on thin-section CT images significantly contributes to its prognosis [2–5]. Therefore, the solid size in a nodule has been adopted as a T factor in the recent TNM staging. However, several recent studies have shown that solid nodules have a worse prognosis than part-solid nodules with ground-glass opacity, even if the solid sizes of tumors are the same [6–10]. For resectable lung cancers, preoperative diagnostic imaging is essential for determining the best therapeutic strategy, including the type of surgical procedure. Therefore, there is a need for imaging techniques that can predict the postoperative prognosis of small-sized solid lung cancers.

A dual-energy CT (DE-CT) technique using two types of tube voltages can enable quantification of the iodine-related attenuation (IRA) of iodinated contrast material in the tumor after intravenous injection without the need for an additional non-contrast CT scan [11–14], thereby reducing radiation exposure during a CT examination. Several research findings indicate that iodine uptake in the arterial phase on contrast CT of primary lung cancer may be associated with some aspects of tumor histopathology such as angiogenesis, differentiation grade, hypoxic cells, and tumor invasiveness [12, 15–18]. Moreover, a few studies indicated that iodine uptake of lung cancer correlated with tumor recurrence after radiotherapy and chemoradiotherapy [19, 20]. Therefore, we speculated that the three-dimensional iodine-related attenuation (3D-IRA) of solid nodules might be associated with the postoperative recurrence of small-sized lung cancers. If that is true, a dual-energy technique would enable preoperative prediction of the prognosis. To date, no study has investigated the correlation between contrast-enhanced dual-energy CT findings and postoperative recurrence in lung cancer.

In this study, we reviewed preoperative data from contrast CT with dual-energy scanning as well as postoperative pathological TNM staging and disease-free survival of resected of lung cancers ≤3 cm, and validated the hypothesis that the 3D-IRA of small-sized solid lung cancers could be a predictive factor for the prognosis.

## Methods and materials

### Patient selection

**Fig. 1:**
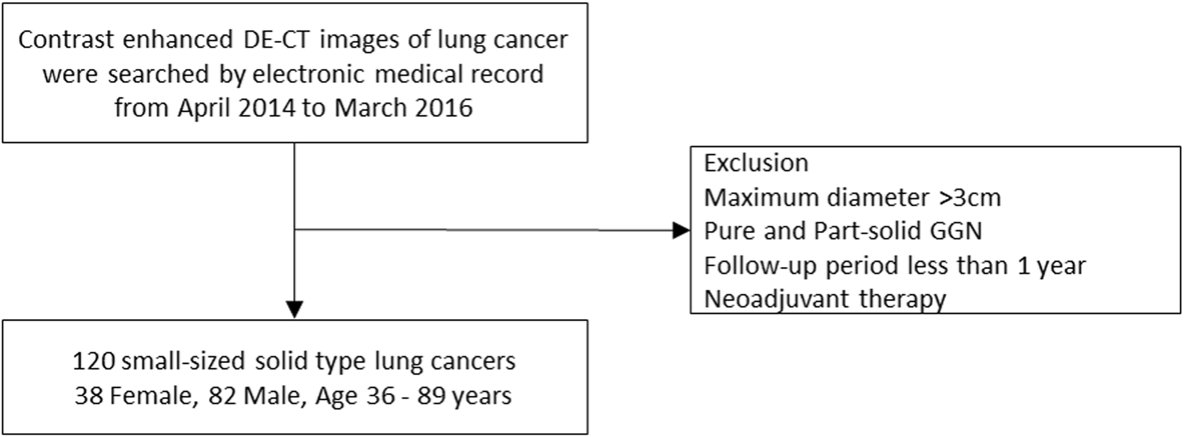
Study cohort flowchart. After applying the exclusion criteria, 120 small-sized (pathological tumor size ≤3 cm in diameter) solid-type lung cancers were included in the present analysis

We reviewed the medical records, postoperative pathological records, and preoperative DE-CT images of patients who underwent surgical lung resection for primary lung cancer (adenocarcinoma, squamous cell carcinomas, adenosquamous carcinomas, large cell neuroendocrine carcinomas, small cell carcinoma, pleomorphic carcinoma, and lymphoepithelioma-like carcinoma) at our hospital between April 2014 and March 2016 (Fig. 1). We limited tumors ≤3 cm in diameter that had been definitively diagnosed by pathological examination after surgical resection. Pure and part-solid ground-glass nodules were excluded. Patients who received neoadjuvant chemoradiotherapy were also excluded. We recorded patient characteristics (gender, age, and body weight) from medical records, and tumor characteristics (pathological tumor size [pSize], histological type, differentiation grade, pathological TNM staging [pStage]) from pathological records. Herein, the seventh edition of union for international cancer control (UICC) TNM staging was used. Cases that were recurrence-free and had a postoperative follow-up period of less than 1 year were excluded. The diagnosis of recurrence was made by thoracic surgeons through routine check-ups by tumor markers, chest X-ray, chest and abdomen CT, and head MRI. PET/CT and biopsies were added to determine the eventual diagnosis in cases of suspected recurrence.

### CT scan protocol

All CT scans were performed with a dual-source CT scanner (SOMATOM Definition Flash; Siemens Healthcare, Tokyo, Japan) in the craniocaudal direction with inspiratory apnea. For vessel enhancement, 96 mL of non-ionic contrast medium (Proscope 300, 300 mgI/mL iopromide, Alfresa Pharma, Osaka, Japan; Optiray 320, 320 mgI/mL ioversol, Tyco Healthcare, Tokyo, Japan; or iopamiron 370, 370 mgI/mL iopamidol, Bayer Healthcare, Tokyo, Japan) was used with flow rates of 4.0 mL/sec. Iopromide was used for patients with body weights < 40 kg, ioversol was used for patients with body weights 40–59 kg, and iopamidol was used for patients with body weights ≥60 kg. Injections were immediately followed by a saline chaser bolus of 20 mL at the same flow rate using a dual-barrel power injector (Dual Shot GX7; Nemoto Kyorindo, Tokyo, Japan).

At our institution, dual-phase contrast-enhanced CTs were performed routinely for preoperative evaluation of lung cancer. The early-phase thoracic CT images were scanned for preoperative evaluation of pulmonary vessels by 3D pulmonary angiography. The scan delay was evaluated by an automatic bolus tracking system with a circular ROI localized on the thoracic aorta. Scanning started automatically as the attenuation of the ROI increased by 70 HU from baseline (about 20–25 s after the start of injection). A dual-energy scan system (tube A at a peak voltage of 140 kVp and tube B at a peak voltage of 80 kVp) was used for both scans with collimation of 64 × 0.6 mm and gantry rotation speed of 285 ms. Data were reconstructed with a slice thickness of 1.0 mm at 1.0-mm increments using iterative reconstruction techniques (SAFIRE, Siemens Healthcare, Tokyo, Japan). Additionally, delayed-phase thoraco-abdominal CT scans were obtained to evaluate lymph node metastasis and abdominal distant metastasis about 2 min after contrast medium injection.

### Analysis of dual-energy CT images

The 3D-IRA of the primary lesion was measured using the syngo “lung nodules” application accessory software (Siemens Healthcare, Tokyo, Japan). The DE-CT technique can create iodine-enhanced images from DE-CT raw data sets of 140 kVp and 80 kVp, and provide absolute measurements of iodine within the tumors. This application can automatically three-dimensionally segment a pulmonary nodule and calculate the mean IRA of pulmonary nodules with a three-material decomposition algorithm. A chest radiologist with 21 years of experience in reading thoracic CT scans segmented the primary tumor and measured the 3D-IRA in the early and delayed phases.

Additionally, corrected 3D-IRA values to reduce the effect of body weight and iodine concentration of contrast medium were calculated as follows:

$$ \mathrm{Corrected}\ 3\mathrm{D}-\mathrm{IRA}=\frac{raw\  data\ of\ 3D- IRA}{Concentration\ of\ contrast\ medium\ \left[\mathrm{mgI}\right]/ Body\ weight\ \left[\mathrm{kg}\right]} $$

### Statistical analysis

First, recurrent and non-recurrent tumors were compared. Gender, histopathological type, and pStage, were compared by chi-squared test. The age, body weight, clinical and pathological tumor size, 3D-IRA, and corrected 3D-IRA were compared by Welch’s t-test. Second, correlations between tumor characteristics and pStage IB-III were individually evaluated using univariate logistic regression analysis. Third, areas under the curve of receiver operating characteristic (ROC) analysis for postoperative recurrence were compared with respect to age, corrected 3D-IRAs, and pSize. Then, Youden’s index was used to determine a cutoff level that would indicate recurrence.

Finally, using the cutoff value of 3D-IRA from the ROC analysis, the patients were classified into two groups; disease-free survival (DFS) was assessed using the Kaplan–Meier method; and the survival curves for each group were compared using the log-rank test. DFS was defined as the interval between the surgery and the first disease recurrence, including local recurrence and distant metastasis, or death from any cause.

Analyses were performed using commercial statistical software: Excel 2013 (Microsoft Corp., Redmond, WA) and a statistical add-in (BellCurve version 3.20; Social Survey Research Information Corp, Tokyo, Japan), and SPSS version 23 (IBM Corp., Amonk, NY). A *p*-value < 0.05 was considered statistically significant.

## Results

A total of 132 resected solid-type lung cancers ≤3 cm in diameter were reviewed. One lesion was excluded because pleural dissemination was noted at the time of surgery. Four recurrence-free lesions were excluded because the follow-up period was shorter than 1 year. Five patients had two and one patient had three synchronous multiple lung cancers. For these patients, the tumor with the highest pathological stage was considered for evaluation, and the remaining seven lesions were excluded.

Finally, 120 consecutive primary lung cancer lesions from 120 patients were selected for analysis (82 males and 38 females; mean age, 67 years [range, 36–89 years]; mean body weight, 58.2 kg [range, 37–85 kg]; mean pSize, 20.5 mm [range, 8–30 mm]). Of these lesions, 77 were adenocarcinomas, 31 were squamous cell carcinomas, four were adenosquamous carcinomas, and two each were large cell neuroendocrine carcinomas, small cell carcinomas, pleomorphic carcinomas, and lymphoepithelioma-like carcinomas. All patients underwent preoperative contrast-enhanced CT and PET/CT staging, and nodal staging was confirmed by endobronchial ultrasound-guided trans-bronchial needle aspiration (EBUS-TBNA) in cases of suspected mediastinal lymph node metastases. In the clinical TNM staging [cStage], 71 tumors were categorized as stage IA, 39 as stage IB, 7 as stage IIA, 1 as IIB, and 2 as IIIA. The surgical procedure was lobectomy in 105 tumors, segmentectomy in 13 tumors and wide wedge resection in 2 tumors. The mean period from CT scan to surgery was 29 ± 18 days. In the pStage, 58 tumors were categorized as stage IA, 30 as stage IB, 9 as stage IIA, 7 as IIB, 15 as IIIA, and 1 as IIIB. Postoperative adjuvant chemotherapy was administered to 66 patients and not to 26, but the status for the remaining 26 patients was unknown. The median postoperative observation period was 47 months, with a range of 5–68 months. During the observation period, 32 patients showed postoperative recurrence, and five patients died of diseases other than lung cancer. The median postoperative recurrence interval was 16.5 months, and the range was 5–59 months.

Recurrent tumors had significantly lower 3D-IRA and corrected 3D-IRA at early phase compared to non-recurrent tumors (*p* = 0.046 and *p* = 0.027, respectively; Table 1), while no significant difference was found at the delayed phase (*p* = 0.124 and *p* = 0.059, respectively; Table 1). Recurrent tumors showed a significantly larger size than non-recurrent tumors (*p* < 0.001). In addition, recurrent tumors had a significantly higher rate of pathological stage IB or higher (p < 0.001).

**Table 1:** Comparison of patient and tumor characteristics between the recurrence and non-recurrence groups

	Non-recurrence	Recurrence	p-value
**Male / Female (n)**	56 / 32	26 / 6	0.067
**Age (years)**	68 ± 9	67 ± 10	0.691
**Body weight (kg)**	58.8 ± 10.3	56.4 ± 9.2	0.217
**pSize (mm)**	19.2 ± 5.7	24.2 ± 5.7	< 0.001
**3D-IRA at early phase (HU)**	36.7 ± 19.1	30.0 ± 14.6	0.046
**Corrected 3D-IRA at early phase**	6.18 ± 3.25	4.94 ± 2.42	0.027
**3D-IRA at delayed phase (HU)**	45.5 ± 15.7	40.6 ± 14.8	0.124
**Corrected 3D-IRA at delayed phase**	7.63 ± 2.51	6.68 ± 2.37	0.059
**Adenocarcinoma / SqCC / Others (n)**	58 / 22 / 8	19 / 9 / 4	0.772
**Differentiation grade 1 / 2 / 3 / 4 (n)**	14 / 53 / 17 / 4	4 / 20 / 6 / 2	0.952
**cStage IA / IB/ II-III (n)**	60 / 24 / 4	11 / 15 / 6	0.002
**pStage IA / IB / II-III (n)**	51 / 24 / 13	7 / 5 / 20	< 0.001
**Lobectomy / Segmentectomy /WWR**	78 / 9 / 1	27 / 4 / 1	0.699
**Adjuvant therapy none / Available (n)**	53 / 15	13 / 13	0.008

*3D-IRA* three-dimensional iodine-related attenuation; *cStage* clinical TNM stage; *pSize* pathological tumor size; *pStage* pathological TNM stage; *SqCC* squamous cell carcinoma, *WWR* wide wedge resection

The mean ± standard deviation (SD) corrected 3D-IRA at early phase were 6.68 ± 3.50 for stage IA, 4.64 ± 2.47 for stage IB, and 5.43 ± 2.35 for stage II-III tumors, and 3D-IRA for stage IA tumors was significantly higher than those for stage IB and stage II-III tumors (*p* = 0.002 and *p* = 0.034, respectively). The mean ± SD corrected 3D-IRA at early phase were 7.79 ± 2.81 for differentiation grade 1 tumors, 5.79 ± 3.04 for grade 2 tumors, 4.97 ± 3.00 for grade 3 tumors, and 4.22 ± 2.74 for grade 4 tumors, and an analysis of variance showed a statistically significant difference among them (*p* = 0.012).

The corrected 3D-IRA at early phase and the tumor size were significantly correlated with stage IB or higher by logistic regression analysis (odds ratio [OR] = 0.817, *p* = 0.003; and OR = 1.123, *p* < 0.001; respectively, Table 2).

**Table 2:** Univariate logistic regression analysis of clinical and pathological factors for pathological stage IB-III

	OR (95% CI)	p-value
**Clinical factors**		
Male vs. Female	1.287 (0.595–2.780)	0.522
Body weight	1.007 (0.972–1.044)	0.706
Age	0.988 (0.950–1.027)	0.528
Corrected 3D-IRA at early phase	0.817 (0.716–0.933)	0.003
Corrected 3D-IRA at delayed phase	0.932 (0.806–1.078)	0.344
**Pathological factors**		
Others vs. Adenocarcinoma	0.838 (0.397–1.769)	0.643
pSize	1.123 (1.052–1.200)	< 0.001
Differentiation grade	1.505 (0.899–2.521)	0.120

*3D-IRA* three-dimensional iodine-related attenuation; *CI* confidence interval; *OR* odds ratio; *pSize* pathological tumor size

**Fig. 2:**
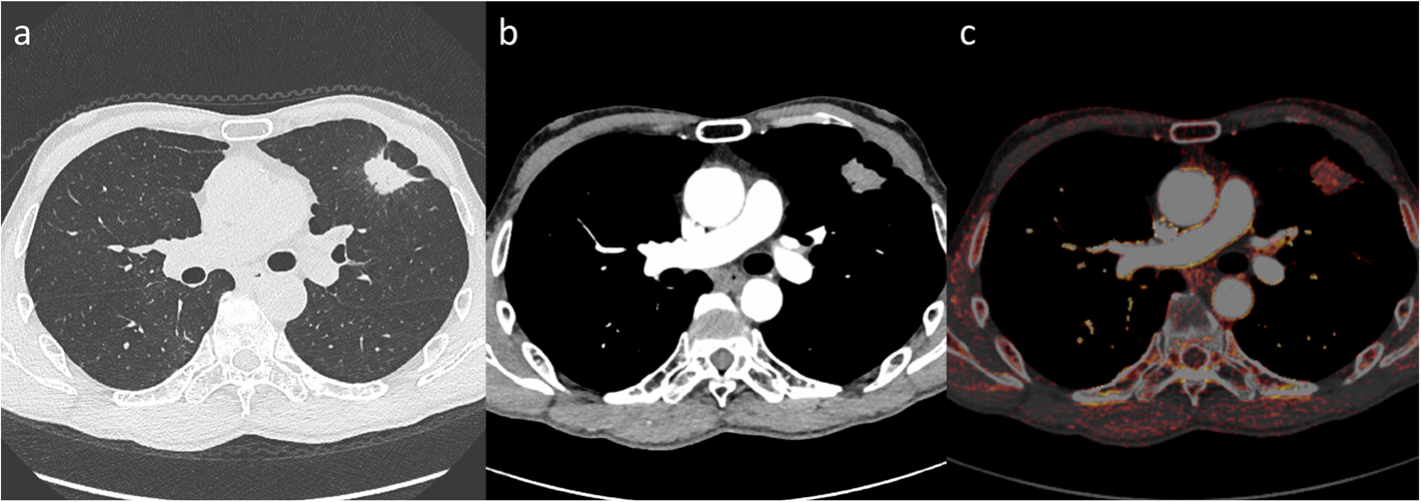
A 68-year-old male patient. An adenocarcinoma (pT2aN0) is observed in the left upper lobe. **a** Lung window setting. **b** Mediastinal window setting at the early phase of contrast-enhanced CT. **c** Iodine-enhanced image in contrast-enhanced CT. The 3D-IRA is 30 HU, and the corrected 3D-IRA is 4.59. The patient developed recurrence of adrenal metastases at 15 months after a left upper lobectomy

**Fig. 3:**
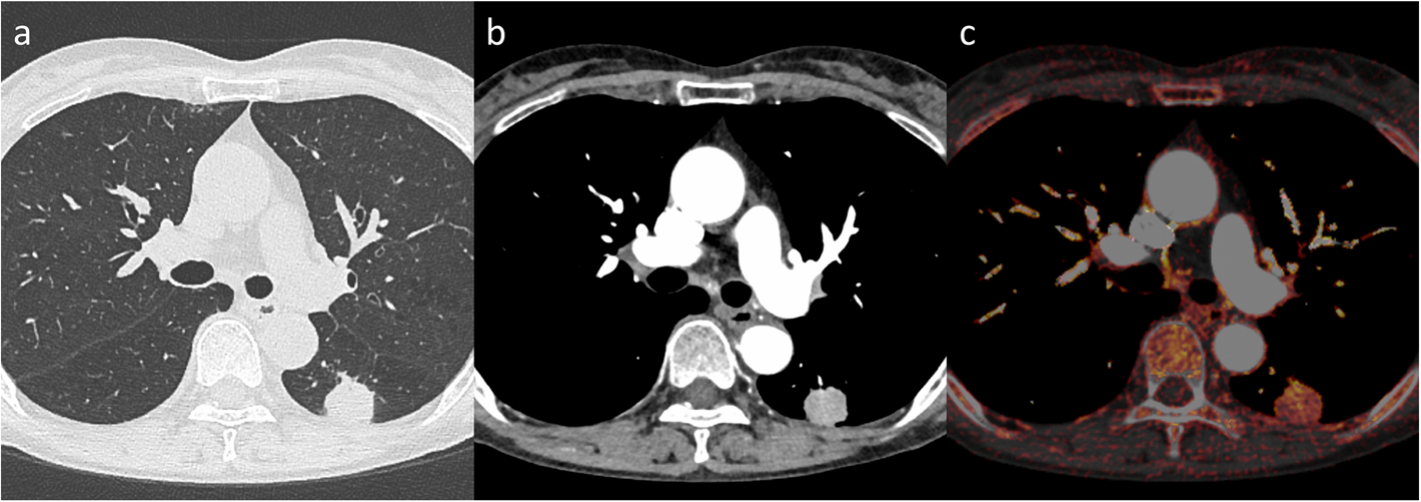
A 75-year-old female patient. A squamous cell carcinoma (pT1bN0) is observed in the left lower lobe. **a** Lung window setting. **b** Mediastinal window setting at the early phase of contrast-enhanced CT. **c** Iodine-enhanced image in contrast-enhanced CT. The 3D-IRA is 48 HU, and the corrected 3D-IRA is 6.45. The patient was recurrence-free for 51 months after a left lower lobectomy

**Fig. 4:**
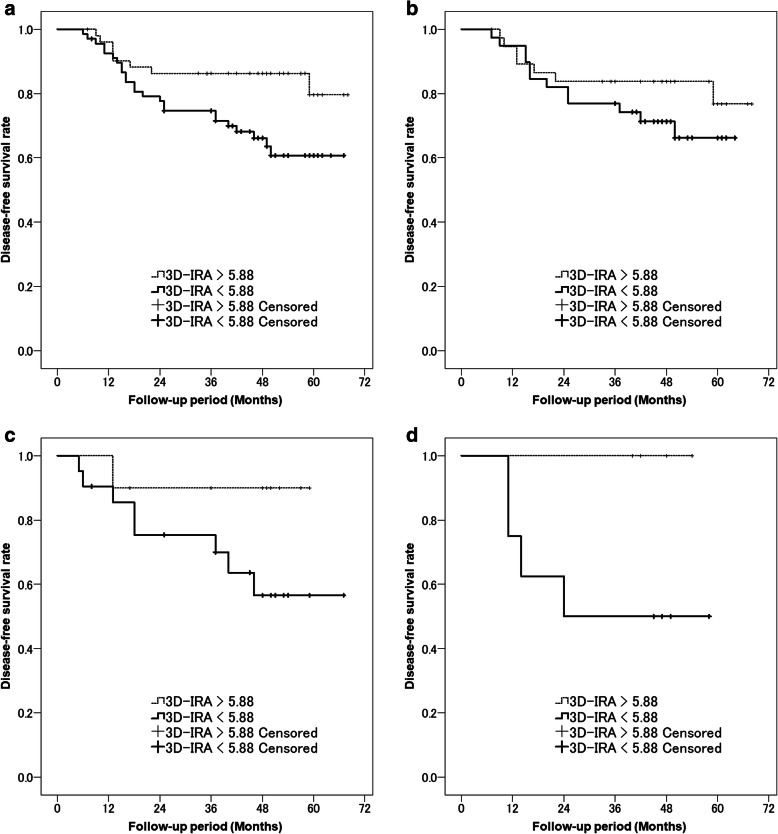
The graph shows Kaplan–Meier curves for disease-free survival according to the corrected 3D-IRA at the early phase of contrast-enhanced CT. **a** Patients with tumors showing 3D-IRA > 5.88 had a significantly better prognosis than those with tumors showing 3D-IRA < 5.88 (*p* = 0.017). **b** Patients with adenocarcinomas, **c** patients with squamous cell carcinomas, **d** patients with other subtype tumors showing 3D-IRA > 5.88 had a better prognosis than those with tumors showing 3D-IRA < 5.88 although there was no significant difference (p = 0.224, p = 0.126, and p = 0.109, respectively)

The area under the curve (AUC) of ROC analysis for postoperative recurrence was 0.624 for the corrected 3D-IRA at early phase (*p* = 0.025), and the suitable cutoff value was 5.88 with a sensitivity of 75.0% and specificity of 50.0% (Table 3). When the patients were divided into two groups using this cutoff value, the tumors with corrected 3D-IRA < 5.88 (Fig. 2) had a significantly poorer prognosis than those with 3D-IRA > 5.88 (Fig. 3) on the Kaplan–Meier survival curve of DFS (mean, 50 months vs. 60 months, *p* = 0.017; Fig. 4a). Patients with adenocarcinomas, those with squamous cell carcinomas, and those with other subtype tumors showing 3D-IRA > 5.88 had a better prognosis than those with tumors showing 3D-IRA < 5.88, although there was no significant difference (*p* = 0.224, *p* = 0.126, and *p* = 0.109, respectively; Fig. 4b-d,).

**Table 3:** Receiver operating characteristic (ROC) analysis for postoperative recurrence

Factors	AUC (95% CI)	p-value	Cutoff value	Sensitivity	Specificity
**Age**	0.520 (0.402–0.639)	0.738	72	0.531	0.648
**Corrected 3D-IRA at early phase**	0.624 (0.519–0.732)	0.025	5.88	0.750	0.500
**Corrected 3D-IRA at delayed phase**	0.598 (0.482–0.715)	0.097	5.51	0.375	0.841
**pSize**	0.772 (0.635–0.848)	< 0.001	25	0.625	0.795

*3D-IRA* three-dimensional iodine-related attenuation; *CI* confidence interval; *pSize* pathological tumor size

## Discussion

It is difficult to preoperatively predict the prognosis of solid-type lung cancers not containing ground-glass opacity. Small-sized lung cancers less than 3 cm, equivalent to T1 in TNM classification, have a good prognosis, although solid-type tumors often recur [6–10]. This study was designed to assess the role of contrast-enhanced DE-CT in predicting the postoperative prognosis of small-sized solid lung cancers resected surgically. We researched the following points: (1) Is the 3D-IRA calculated by DE-CT associated with the TNM staging, even in solid-type tumors ≤3 cm in diameter? (2) Are the 3D-IRAs associated with their postoperative DFS? This study revealed that the 3D-IRA at early phase was significantly correlated with pathological TNM staging. The low-3D-IRA tumors tended to show higher TNM stage. In addition, 3D-IRA at early phase was correlated with postoperative recurrence; tumors with a low 3D-IRA had significantly shorter DFS than tumors with high 3D-IRA.

The tumors with low 3D-IRA tended to show higher TNM stage because of pleural invasion, chest wall invasion, intrapulmonary metastasis, and lymph node metastases. The stage IB-III tumors showed significantly lower 3D-IRA at the early phase than the stage IA tumors. In addition, logistic regression analysis revealed that tumor size and corrected 3D-IRA at early phase were significant factors for stage IB or higher tumors. A few previous studies have shown that the 3D-IRA significantly correlates with the locoregional invasiveness of lung cancers, which is consistent with the current results [15, 16]. It is presumed that the strong invasiveness of the tumor will lead to postoperative recurrence even after complete resection. The recurrent tumors included a high rate of stage IB or higher tumors, and these showed significantly lower 3D-IRA than the non-recurrent tumors.

The ROC analysis indicated that the suitable cutoff value of corrected 3D-IRA at early phase was 5.88 for recurrence. This value represents 37 HU for a patient weighing 50 kg with a contrast media dose of 320 mgI and 36 HU for a patient weighing 60 kg with a contrast media dose of 370 mgI. In previous literature examining 3D-IRA and tumor differentiation, these values correspond to Grade 2 and Grade 3 boundary values [12]. Therefore, tumors with corrected 3D-IRA < 5.88 might include many poorly or undifferentiated carcinomas. When this cutoff value was used to divide the patients into two groups, 3D-IRA < 5.88 showed significantly poorer prognosis on the Kaplan–Meier survival curve. These curves indicate that 3D-IRA has a significant impact on tumor recurrence after 1 year post-surgery. We examined Kaplan-Meier survival curves for each pathological subtype, and for all subtypes, 3D-IRA < 5.88 tumors showed a poor prognosis.

In recent years, stereotactic radiotherapy (SRT) has been increasingly used as an alternative to surgery for small-sized lung cancer. The weakness of SRT is that the histopathological invasiveness of the tumor cannot be determined surgically. For small-sized lung tumors, it is sometimes difficult to determine the histopathology using transbronchial lung biopsy and CT-guided transcutaneous needle biopsy. Therefore, it is more important to predict the invasiveness and prognosis of the tumor by radiological imaging before treatment. One previous study reported that the IRA at the early phase of contrast DE-CT is useful in predicting recurrence after SRT [19]. Although surgery and SRT are different treatment modalities, our results are consistent with their conclusions. Therefore, it is presumed that IRA at the early phase reflects the invasiveness and grade of the tumor rather than the responsiveness of the tumor to radiation therapy.

This study has several limitations. First, it was a retrospective and a single-center study. Second, we were not able to examine postoperative adjuvant therapy in detail in patients transferred to the hospital. However, adjuvant chemotherapy might not substantially affect our results because it was performed significantly more cases in the recurrence group. Finally, in this retrospective study, we were not able to examine the relationship between 3D-IRA and pathological genetic prognostic markers such as Ki-67 and EGFR mutation [21–23].

## Conclusions

The 3D-IRA of small-sized solid lung cancers measured by contrast-enhanced DE-CT was significantly associated with pathological invasiveness and postoperative recurrence. Tumors with a low 3D-IRA at the early phase tended to have a higher TNM stage and a significantly poorer prognosis.

## Data Availability

Not applicable.

## Abbreviations

3D-IRA: Three-dimensional iodine-related attenuation
AUC: Area under the curve
cStage: clinical TNM staging
DE-CT: Dual-energy computed tomography
EBUS-TBNA: Endobronchial ultrasound-guided trans-bronchial needle aspiration
pSize: pathological tumor size
pStage: pathological TNM staging
ROI: Region of interest
ROC: Receiver operating characteristic
SqCC: Squamous cell carcinoma
WWR: Wide wedge resection
